# Comparison of a Reduced Needle With a Thin Tip and a Standard Thin Needle (Same Width Overall) for Oocyte Retrieval

**DOI:** 10.7759/cureus.62787

**Published:** 2024-06-20

**Authors:** Akira Nakabayashi, Yuriko Hashimoto, Yu Horibe, Tomomi Hashimoto, Shuko Murata, Jun Kakogawa

**Affiliations:** 1 Obstetrics and Gynecology, Tokyo Women's Medical University, Tokyo, JPN; 2 Gynecology, Tokyo Women's Medical University, Tokyo, JPN

**Keywords:** ivf, thin needle, oocyte retrieval, reduced needle, needle diameter

## Abstract

Introduction: At our facility, oocyte retrieval had previously been performed with a 20-gauge standard needle that is uniformly thin overall (tSN); but recently, we have instead started using reduced needles, with a 20-gauge tip and 17-gauge body (RN). Until now, there have been comparisons between RN and thick standard needles, but there have been no comparisons between RN and tSN. The purpose of this study was to compare oocyte retrieval outcomes using RN with tSN.

Methodology: Information on oocyte retrieval was extracted from the medical records of 304 cycles performed at our facility from January 2020 to December 2023. The oocyte retrieval outcomes of the two types of needles were compared retrospectively with respect to age, anti-Müllerian hormone (AMH), procedure time, additional sedatives, number of follicles punctured, number of oocytes retrieved, number of oocytes fertilized, oocyte recovery rate, and fertilization rate.

Results: When AMH ≥ 1.2 ng/mL, the procedure time was 9.3 ± 3.7 and 12.1 ± 4.6 minutes in the RN and tSN groups, respectively (*P *< 0.001), and the need for additional sedatives was also significantly different: 54.0% in the RN group and 78.5% in the tSN group (*P *= 0.002). The oocyte recovery rate was significantly different between the RN and tSN groups at 65.3% and 61.2%, respectively (*P *= 0.046), and the fertilization rate was significantly different between the RN and tSN groups at 56.8% and 66.8%, respectively (*P *< 0.001). There were no significant differences by age, AMH, number of follicles punctured, number of oocytes retrieved, or number of oocytes fertilized.

Conclusions: Without diminished ovarian reserve, RN reduced procedure time and the need for additional sedatives compared to tSN. In addition, the number of oocytes fertilized per oocyte retrieval remained the same, indicating that oocyte retrieval performance was not affected.

## Introduction

Transvaginal ultrasound-guided oocyte retrieval was first reported in 1985 [1] and is now a standard procedure. Live birth rates increase with an increasing number of retrieved oocytes up to 15 [2], and, commonly, ovarian stimulation is performed to obtain as many oocytes as possible. Since appropriate analgesia is necessary for the pain associated with oocyte retrieval, oocyte retrieval is performed under local anesthesia or conscious sedation [3]. The size of the needle used for oocyte retrieval is one factor affecting pain, and it is speculated that the thinner the needle, the less pain is experienced. A few prospective studies have attempted to optimize needle size during oocyte retrieval. Reducing the thickness of the needle from 15 gauge to 17 or 18 gauge has previously been shown to reduce pain, without affecting the number of oocytes collected, their quality, or clinical pregnancy rates [4-6]. Recently, a prospective pilot study on further miniaturization was conducted, and while a 20-gauge needle did not affect the oocyte yield compared to a 17-gauge needle, it did prolong the procedure time [7]. However, thin needles bend more easily, which can cause the needle tip to shift out of the puncture guide.

Recently, reduced needles have been available, in which only the tip of the needle that punctures the tissue is reduced in diameter. The 20-gauge tip is thinner than the 17-gauge body. The tip is thinned to reduce bleeding and pain, while the body remains thick to maintain the rigidity of the needle for accurate puncture, to maintain the suction flow rate, not to prolong the procedure time. A randomized controlled trial (RCT) showed that oocyte aspiration performed with the reduced needle with a 20-gauge tip resulted in significantly less overall pain and less vaginal bleeding, without prolonging the procedure time or influencing the oocyte recovery rate, when compared with a 17-gauge standard needle [8]. In addition, a similar RCT was conducted at a single center and showed that the reduced needle with a 20-gauge tip lowered pain scores during oocyte retrieval, and they remained lower up until two days after the procedure, compared to 16-gauge standard needles [9].

All previous reports have compared needle sizes at the tissue puncture sites and have shown no difference in oocyte retrieval outcomes and decreased pain with thinner needles, whether the needle was standard or reduced. On the other hand, there has been no comparison between needles that have a thin tip and different-sized body portions. At our facility, 20-gauge standard needles that are uniformly thin overall (tSN) were used in the past, but since 2022, 20-/17-gauge reduced needles with a thin tip (RN) have been used instead. The objective of this study is to examine the effect of different thicknesses of the needle body on oocyte retrieval.

## Materials and methods

Information on oocyte retrieval was extracted from the medical records of 304 cycles in which oocytes were retrieved at our facility from January 2020 to December 2023. No restrictions were set for age or anti-Müllerian hormone (AMH) level, and all cycles were included with no exclusions. The data to be extracted were age, AMH, start time, end time, sedation medication, number of follicles punctured, number of oocytes obtained, number of oocytes fertilized, and type of needle. The study was approved by the University Ethics Committee of Tokyo Women’s Medical University (approval number 5704).

Comparative analysis was performed by dividing the group into two groups, one with expected normal ovarian response (AMH ≥1.2 ng/mL) and one with expected poor ovarian response (AMH < 1.2 ng/mL); and in each, the oocyte retrieval outcomes of the two types of needles with respect to age, AMH, procedure time, additional sedative, number of punctured follicles, number of oocytes obtained, number of oocytes fertilized, oocyte recovery rate, and fertilization rate were compared. The procedure time was defined as the time from the start of vaginal cleansing to the time after the puncture when hemostasis was confirmed and the vaginal speculum was removed. The oocyte recovery rate was calculated by dividing the total number of oocytes obtained by the total number of follicles punctured, and the fertilization rate was calculated by dividing the total number of oocytes fertilized by the total number of oocytes obtained. For comparison between the two groups, the Mann-Whitney U-test for continuous variables and Fisher’s exact test were used for dichotomous variables. All significance tests were two-sided and conducted at the 0.05 significance level. All statistical analyses were performed using statistical software, JMP Pro version 17 (SAS Institute, Cary, NC).

Oocyte retrieval at our facility is mainly performed by two fertility specialists, who puncture even small follicles of about 10 mm. Several oocytes were collected together in round-bottom tubes without distinguishing the follicle size. Puncture needles were either tSN or RN. The tSN is made by Kitazato Corporation (Shizuoka City, Japan), with a total length of 30 cm and an overall specification of a 20-gauge needle (0.9 mm outer diameter). On the other hand, the RN is made by Vitrolife AB (Sweden) and has a total length of 35 cm, with the 5 cm tip having a 20-gauge needle (0.9 mm outer diameter) and the 30 cm body having a 17-gauge needle (1.4 mm outer diameter). The area of the body is 1.54 mm^2^ while the area of the narrower tip is 0.63 mm^2^, 59% smaller. For aspiration of the follicular puncture, a Cook Aspiration Unit (Cook) was used, with a pressure of 230 mmHg for both needles. For sedation, a bolus of 200 mg of sodium thiopental is administered intravenously, with additional doses as needed depending on the patient's pain.

 For patients without diminished ovarian reserve, controlled ovarian hyperstimulation was performed; for patients with diminished ovarian reserve, mild ovarian stimulation was performed. Ovarian stimulation was performed using follicle-stimulating hormone (urinary or recombinant) or human menopausal gonadotrophin in combination with GnRH agonist or antagonist or progestin according to each patient’s status. Dosage also was set individually, taking into account age, AMH level, body weight, and previous oocyte retrieval outcomes. The timing of oocyte retrieval was determined based on follicle development and estradiol levels. HCG injection was administered 34 hours before oocyte retrieval. The decision to use in vitro fertilization (IVF) or intracytoplasmic sperm injection (ICSI) was based on sperm status and previous treatment history.

## Results

From January 2020 to March 2022, tSN was used, and in 2021, RN was used for 15 cycles on a trial basis. In April 2022, the needle was switched to RN only. In total, tSN was used for 148 cycles and the RN for 156 cycles. The cycles for AMH ≥ 1.2 ng/mL were 155, with 87 cycles using the RN, and 68 cycles using tSN. The cycles for AMH < 1.2 ng/mL was 149, with 69 cycles using the RN and 80 cycles using tSN.

The results analyzed by oocyte retrieval are shown in Table 1. There were no significant differences between the RN and tSN groups in terms of patient background, including age and AMH. The procedure time was 9.3 ± 3.7 and 12.1 ± 4.6 minutes in the RN group and the tSN group, respectively, when AMH ≥ 1.2 ng/mL, showing a significant difference (*P *< 0.001). When AMH < 1.2 ng/mL, the mean time to treatment was 6.6 ± 3.2 and 7.9 ± 4.3 minutes, respectively, which were not significantly different (*P *= 0.12). The additional sedative was significantly different in 54% and 78.5% of the RN and tSN groups, respectively, when AMH ≥ 1.2 ng/mL (*P *= 0.002); and not significantly different in 23.6% and 12.5% when AMH < 1.2 ng/mL, respectively (*P *= 0.09). No significant difference was found in the number of follicles punctured per oocyte retrieval. The number of oocytes obtained was 10.1 ± 7.6 and 8.3 ± 5.4 in the RN and tSN groups, respectively, when AMH ≥ 1.2 ng/mL (*P *= 0.22), and 2.9 ± 2.6 and 1.8 ± 1.6 when AMH < 1.2 ng/mL, respectively (*P *= 0.009). The number of oocytes fertilized was not significantly different between the two groups (*P *= 0.08).

**Table 1:** Baseline characteristics and oocyte retrieval procedure data in each group. AMH, anti-Müllerian hormone; RN, reduced needle; tSN, thin standard needle

	AMH ≥ 1.2			AMH < 1.2		
	RN	tSN	*P*-value	RN	tSN	*P*-value
Cycles (*n*)	87	68		69	80	
Age (years)	37.9 ± 4.4	37.9 ± 3.9	0.77	41.7 ± 3.2	42.7 ± 2.60	0.11
AMH (ng/mL)	3.8 ± 2.5	4.1 ± 3.1	0.81	0.45 ± 0.23	0.51 ± 0.31	0.06
Procedure time (minutes)	9.3 ± 3.7	12.1 ± 4.6	<0.001	6.6 ± 3.2	7.9 ± 4.3	0.12
Additional sedatives (%)	54.0	78.5	0.002	23.6	12.5	0.09
Follicles punctured (*n*)	15.4 ± 9.7	13.6 ± 7.6	0.31	4.5 ± 2.9	3.6 ± 2.3	0.09
Oocytes retrieved (*n*)	10.1 ± 7.6	8.3 ± 5.4	0.22	2.9 ± 2.6	1.8 ± 1.6	0.009
Oocytes fertilized (*n*)	5.7 ± 5.2	5.5 ± 4.7	0.97	1.6 ± 1.6	1.2 ± 1.3	0.08

The results analyzed by follicle and oocyte are shown in Table 2. The oocyte recovery rate was significantly different between the RN and SN groups at 65.3% (877/1,343) and 61.2% (564/922) when AMH ≥ 1.2 ng/mL (*P *= 0.046) and 65.3% (203/311) and 49.8% (145/291) when AMH < 1.2 ng/mL (*P* < 0.001). Fertilization rates were significantly different between the RN and tSN groups when AMH ≥ 1.2 ng/mL in 56.8% (498/877) and 66.8% (377/564), respectively (*P *< 0.001), and not significantly different when AMH < 1.2 ng/mL in 55.7% (113/203) and 63.4% (92/145), respectively (*P *= 0.15).

**Table 2:** Oocyte recovery rates and fertilization rates. AMH, anti-Müllerian hormone; RN, reduced needle; tSN, thin standard needle

	AMH ≥ 1.2			AMH < 1.2			
	RN	tSN	*P*-value	RN	tSN	*P*-value	
Oocyte recovery rate (%)	65.3	61.2	0.046	65.3	49.8	<0.001	
Total follicles punctured (*n*)	1,343	922		311	291		
Total oocytes retrieved (*n*)	877	564		203	145		
Fertilization rate (%)	56.8	66.8	<0.001	55.7	63.5	0.15	
Total oocytes retrieved (*n*)	877	564		203	145		
Total oocytes fertilized (*n*)	498	377		113	92		

## Discussion

The number of follicles punctured in cycles with AMH < 1.2 ng/mL was small, and no significant difference was found in the procedure time required between the two types of needles. The number of follicles punctured in cycles with AMH > 1.2 ng/mL was 10 or more, and the RN group had a shorter procedure time of approximately 3 minutes than the tSN group. The final number of oocytes fertilized was not significantly different between the two types of needles, regardless of AMH value; so the change to RN had no adverse effect on oocyte retrieval outcomes. The oocyte recovery rate was in the 60% range, with a low rate of 50% in the tSN group with AMH < 1.2 ng/mL. The fertilization rate was also lower in the RN group and approximately 10% lower in the AMH ≥ 1.2 ng/mL group.　

In conducting this comparison, to approximate the number of follicles punctured between the two groups being compared, we analyzed the outcomes separately for AMH ≥ 1.2 and <1.2 ng/mL, which is used in the POSEIDON criteria for predicting poor ovarian response (POR) [10]. Regarding the number of follicles punctured, a difference of about 10 was observed between AMH ≥ 1.2 and <1.2 ng/mL. The mean age at AMH < 1.2 ng/mL was 41.7 years in the RN group and 42.7 years in the tSN group, and as a result, the number of oocytes fertilized was less than 2; about half of the cycles performed at our facility corresponded to this age group, so we used this age group for comparison. The high average age at which IVF is performed is characteristic of Japan [11] and is attributed to late marriages and the fact that egg donation is not common [12-13]. Even in the previous reports comparing the RN with the thick standard needle, the average age of patients was 34 years in Wikland et al.'s report [8] and 33 years in Buisman et al.'s report [9], which is younger than the 37.9 years at AMH ≥ 1.2 ng/mL in this comparison.

In the present study, the RN reduced the procedure time compared to the tSN when AMH ≥ 1.2 ng/mL, a result consistent with previous reports. Factors contributing to the shorter procedure time were differences in flow rate and ease of use. The flow rate is determined by the inner diameter of the needle and the suction pressure, and to maintain a constant flow rate, a high negative pressure is required when the needle is thin. When we used the tSN, oocyte retrieval was performed at the manufacturer's recommended pressure of 230 mmHg; the RN procedure was also done at the same pressure, so the difference in flow rate was one of the factors that shortened the procedure time. Because aspiration vacuum pressure influences oocyte retrieval [14], whether to lower the pressure with the RN is an issue for the future.

There was no significant difference in the number of oocytes fertilized (5.7 ± 5.2 and 5.5 ± 4.7 in the RN and tSN groups, respectively) when AMH ≥ 1.2 ng/mL, consistent with previous reports that showed no difference in oocyte retrieval outcomes due to needle differences. The low oocyte recovery rate of 50% in the tSN group with AMH < 1.2 ng/mL could be attributed to the high mean age of 42.7 years. The lower fertilization rate in the RN group was initially attributed to the different ratios of small follicles between the two groups. One reason for the lower fertilization rate in the RN group may have been the higher proportion of small follicles. Small follicles of 8-12 mm have been reported to have a lower ratio of M2 oocytes and a lower fertilization rate than moderate follicles of 13-24 mm or larger follicles of 24 mm or more [15]. The results of ICSI performed at AMH ≥ 1.2 ng/mL showed that although the ratio of M2 oocytes to acquired oocytes was 83.7% (401/479) and 83.6% (188/225) in the RN and tSN groups, respectively, with no significant difference (*P *= 1.00), the fertilization rates were 54.3% (260/479) and 64.4% (145/225), with no significant difference (*P *= 0.01); the reason for the lower fertilization rate was not due to the difference in the ratio of immature oocytes. From this verification, it was determined that there was no difference in the ratio of small follicles. Fertilization is influenced by the culture medium used, as well as the quality of sperm and oocytes, in addition to the retrieval method employed. In this comparison, the same culture medium was used, but it remains unclear what caused the lower fertilization rate in the RN group.

A limitation of this study was its retrospective nature, with the timing and methods of oocyte retrieval not being pre-designed. However, since this was a single-center study and the physicians involved in the oocyte retrieval were the same, we believe that there is little significant bias. In addition, the cases were assigned by period, and in such cases, the providers may have differed because the cases were performed at different times. However, the physician who performed the oocyte retrieval and the embryologist who handled the oocytes were the same during the relevant period. However, the impact cannot be ignored, as individual skills improve. Another limitation was the difficulty in comparing oocyte retrieval outcomes. The number of mature oocytes, fertilization rate, cleavage rate, number of embryos transferred, number of embryos frozen, good quality embryo rate, implantation rate, pregnancy rate, and so on, are the items to be evaluated. However, they are greatly influenced by the cause of infertility, the indication for IVF or ICSI, and the condition of the oocytes and sperm. In this study, oocyte retrieval outcomes performed on the same patient were included regardless of the number of times, which may have increased the proportion of patients who did not become pregnant and reflected poor oocyte retrieval outcomes. To reflect the effect of the puncture needle, only the first oocyte retrieval should be included.

Accurate puncture of the follicle requires adjustment of direction and force. If the needle becomes curved, it becomes difficult to adjust direction and force. Therefore, a thicker needle that maintains rigidity is theoretically superior. This comparison was conducted between needles with a thicker body and needles with a thinner body, and we found that the usability of the thinner needle with a thicker body was better in terms of handling. However, we were not able to extract any data on operability, and this is an issue to be addressed in the future.

## Conclusions

This comparison revealed that in cycles without diminished ovarian reserve, the RN reduced the procedure time and the frequency of adding sedation medication compared to tSN. In addition, the number of oocytes fertilized remained the same, indicating that there was no negative impact on oocyte retrieval outcomes. The reduced needle, which reduces pain while maintaining the same oocyte retrieval outcomes as the thick standard needle, also reduces the procedure time while maintaining the same oocyte retrieval outcomes as the thin needle.
